# A Novel Weighted Total Difference Based Image Reconstruction Algorithm for Few-View Computed Tomography

**DOI:** 10.1371/journal.pone.0109345

**Published:** 2014-10-02

**Authors:** Wei Yu, Li Zeng

**Affiliations:** 1 Key Laboratory of Optoelectronic Technology and System of the Education Ministry of China, Chongqing University, Chongqing, China; 2 College of Mathematics and Statistics, Chongqing University, Chongqing, China; 3 Engineering Research Center of Industrial Computed Tomography Nondestructive Testing of the Education Ministry of China, Chongqing University, Chongqing, China; University of Zurich, Switzerland

## Abstract

In practical applications of computed tomography (CT) imaging, due to the risk of high radiation dose imposed on the patients, it is desired that high quality CT images can be accurately reconstructed from limited projection data. While with limited projections, the images reconstructed often suffer severe artifacts and the edges of the objects are blurred. In recent years, the compressed sensing based reconstruction algorithm has attracted major attention for CT reconstruction from a limited number of projections. In this paper, to eliminate the streak artifacts and preserve the edge structure information of the object, we present a novel iterative reconstruction algorithm based on weighted total difference (WTD) minimization, and demonstrate the superior performance of this algorithm. The WTD measure enforces both the sparsity and the directional continuity in the gradient domain, while the conventional total difference (TD) measure simply enforces the gradient sparsity horizontally and vertically. To solve our WTD-based few-view CT reconstruction model, we use the soft-threshold filtering approach. Numerical experiments are performed to validate the efficiency and the feasibility of our algorithm. For a typical slice of FORBILD head phantom, using 40 projections in the experiments, our algorithm outperforms the TD-based algorithm with more than 60% gains in terms of the root-mean-square error (RMSE), normalized root mean square distance (NRMSD) and normalized mean absolute distance (NMAD) measures and with more than 10% gains in terms of the peak signal-to-noise ratio (PSNR) measure. While for the experiments of noisy projections, our algorithm outperforms the TD-based algorithm with more than 15% gains in terms of the RMSE, NRMSD and NMAD measures and with more than 4% gains in terms of the PSNR measure. The experimental results indicate that our algorithm achieves better performance in terms of suppressing streak artifacts and preserving the edge structure information of the object.

## Introduction

As an extremely valuable diagnostic tool, computed tomography (CT) has been widely used in medical area. With this powerful tool, many valuable internal features can be extract without cutting the object [1], [2]. However, during clinical exams, excessive X-ray radiation exposure may increase the lifetime cancer risk [3], [4]. Thus, it has great significance to use shorter time of radiation exposure and lower patient radiation dose to reconstruct numerically accurate tomographic images. To reduce radiation dose, few-view CT has been an important CT imaging modality. In this scanning data situation, tomographic image is reconstructed from the projection data collected by sparse angular sampling [5]–[9]. For few-view CT, due to the projection data obtained is not theoretically sufficient for exact reconstruction of tomographic images, conspicuous streak artifacts are present in reconstructed images by conventional analytic algorithms such as filtered back-projection [5], [10]–[12]. In this paper, we mainly focus the iterative reconstruction algorithm for few-view CT.

Since the development of the large computational capacities in graphical processing unit and the ongoing efforts towards lower doses have made in CT, iterative reconstruction has become a hot topic for all major vendors of clinical CT systems in the past years [13]–[17]. The algebraic reconstruction technique and simultaneous algebraic reconstruction technique (SART) are two classical reconstruction algorithms for CT image reconstruction [18], [19]. Since the projection data are incomplete, using the two algorithms, obvious artifacts and noise are present in reconstructed images. With the development of compressed sensing theory [20]–[22], compressed sensing based iterative reconstruction algorithm has drawn much attention in the medical imaging and other tomographic imaging modalities. By adopting the compressed sensing based iterative reconstruction algorithm, the image can be reconstructed from rather limited projection data [23]. In mathematics, actually, CT image reconstruction with few-view projection data is taken as an ill-posed inverse problem. To solve this problem, regularization method is usually adopted, and corresponding unconstrained optimization problem can be formulated [24]. In the unconstrained optimization problem, the objective function usually contains two terms. The first term is data fidelity term which constraints the data consistency between measured projection data and model data. The second term is regularization term which is designed according to the priori information of the image.

In the CT reconstruction field, it is likely that images are not sparse themselves, but image coefficients in some transform domains show sparsity. In image gradient transform, the L1 norm of the image gradient magnitudes (also known as total variation (TV) of image) are approximately sparse. If 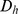 and 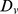 represent the horizontal and vertical gradient operators respectively, then TV regularization term can be expressed as 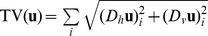. This regularization term which was originally proposed for image denoising [25], has been extended in the field of few-view CT image reconstruction [5]. Subsequently, many other related reconstruction algorithms have been developed [6]–[8], [23], [26].

In 1996, Li and Santosa suggested that total difference (TD) which was defined as 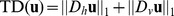, was a reliable and computationally efficient approximation to the TV in image restoration [27]. In 2010, by constructing a pseudo-inverse of the discrete TD, a TD minimization algorithm with soft-threshold filtering (TDM-STF) was developed for few-view CT. It can improve the convergence and efficiency of TV based minimization methods [28]. In the TDM-STF algorithm, TD is taken as the regularization term. Later, the TDM-STF algorithm was applied to a multisource x-ray interior imaging system [29]. In their work, they accelerated the convergence speed of TDM-STF by incorporating a fast iterative shrinkage thresholding algorithm [30]. It shows that when obtaining the same image quality, the TDM-STF may need less iterations and total computational cost for practical application. However, the TD seeks the gradient sparsity horizontally and vertically, but fails to enforce the gradient continuity. Thus, the TD is prone to recovering an image of sharp horizontal and vertical edges. To overcome this shortcoming of TD, a new measure (called weighted total difference (WTD) measure hereafter) was utilized by Shu and Ahuja for compressive sampling [31]. In their work, they proposed a hybrid compressive sampling method for recovering a piecewise smooth image from limited measurements. Since the WTD measure exploits the continuity and sparsity simultaneously in the partial gradient domain, all possible sharper edges of the image can be recovered from limited measurements. In [31], WTD measure was taken as the regularization term. Note that model investigated in [31] is different from the model for CT reconstruction since WTD combines two complementary sampling systems for image recovery. In this work, we consider incorporating WTD measure into the model for CT reconstruction due to the good property of WTD.

With the aim to eliminate the undesired streak artifacts and preserve the edge structure information of the object, in this paper, we propose a novel reconstruction algorithm based on WTD minimization for few-view CT. The proposed reconstruction model combines the CT imaging model and the WTD measure. In the proposed reconstruction model, the WTD measure is taken as the regularization term. The differences between the WTD in current study and in the study [31] lie in the application fields and the corresponding problems need to solve. To solve our model effectively, the soft-threshold filtering (STF) method and a fast iterative shrinkage thresholding algorithm are employed to accelerate the converging speed of our algorithm. For simplicity, in the following sections, we referred to our algorithm as WTDM-STF.

The rest of the paper is organized as follows. In section Method, we illustrate the CT imaging model and describe the WTDM-STF reconstruction algorithm for few-view CT, together with an efficient iterative scheme. Moreover, the data acquisitions and performance evaluations are also outlined in this section. In the following section, numerical results and discussion are presented and conclusions are given in final section.

## Methods

### CT imaging model

The model of fan beam CT imaging can be approximated as following discrete linear system [19]:
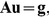
(1)where 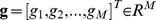 is the measured projection data which can be represented by a vector of size *M*, *M* is the number of the transmission rays, 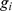 is the ray-sum measured with the 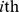 ray, 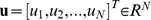 denotes the image to be reconstructed which can be represented by a vector of size *N*, *N* is the number of image pixels. 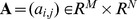 is the system matrix which represents forward projection. The system matrix weight 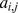 represents the contribution of the 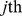 pixel to the 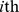 ray-sum. In our experiments, the system matrix weights 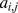 are computed by calculating the intersection length of the 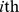 ray through the 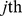 pixel. Given the projection data acquired from the detector, the aim for image reconstruction is to solve the Eq. (1) for 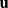 from the measured data 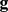. As for few-view CT problem, the number of the measured projection data 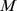 is much smaller than the number of image pixels 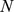, then, the Eq. (1) is underdetermined. Therefore, how to reconstruct approximately accurate CT image is important for practical applications.

### The WTDM-STF algorithm

Inspired by the wok in [31], the WTD measure not only enforces the gradient sparsity horizontally and vertically, but also enforces the gradient continuity. By the WTD measure, the sharp and clear edges can be better preserved than TD. The WTD of image 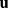 is defined as follows
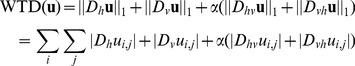
(2)Where 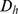, 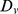, 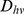 and 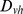 are respectively horizontal, vertical and two diagonal partial gradients operators, i.e., 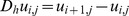, 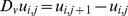, 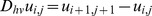, 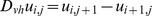. The first two terms measure the gradient sparsity; the last two terms measure the gradient continuity, and the parameter 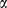 plays a crucial role in balancing the gradient sparsity and the gradient continuity. In this work, we combine the WTD (see also in Eq. (2)) and CT imaging model (see also in Eq. (1)) to formulate a novel reconstruction model for few-view CT. Using 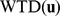 as the regularization term, the developed image reconstruction model can be expressed as



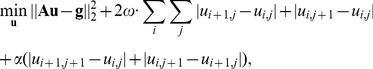
(3)From the model above, the TD-based image reconstruction model [28] is one special case of model (3), which is equivalent to the model (3) when 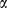 equals to zero. In our work, we fix the parameter 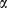 equals to 1.0, which equally penalizes the gradient sparsity and the gradient continuity. The difference between TD-based image reconstruction model and our model lies in the original TD-based regularization just enforces the sparsity horizontally and vertically in the gradient domain, while the sparsity diagonally and the directional continuity of gradients are not considered. Therefore, the sharp and clear edges in the CT image can be well preserved along more directions by our reconstruction algorithm.

To solve the above model in (3), there are many ways, such as soft-threshold filtering (STF) method [32], [33] and Split Bregman method [34]. The STF method, whose convergence and efficiency have been theoretically proven, has already been applied for CT reconstruction in [28], [29]. Similar to [29], in this work, we solve our reconstruction model in the soft-threshold filtering framework. After the soft-threshold filtration, we need to construct a pseudo-inverse of the WTD. The detailed iterative steps of our WTDM-STF algorithm can be summarized as the following:

(1) Initialization:


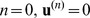
, 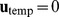, 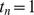, 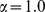.

(2) The data constraint step. Update 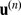 using the SART formula:


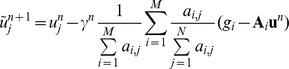
, 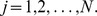
where 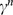 is a relax parameter.

(3) Soft-threshold filtering step, the pseudo-inverse of 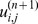 is constructed similar to reference [28],
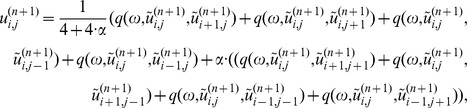
where



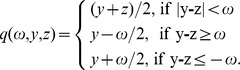



the threshold 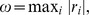 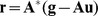 [33].

(4) Acceleration technique:
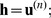


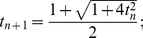


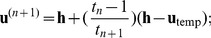


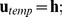



(5) Initialize next loop:



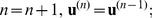



Return to step (2) until the stopping criteria is satisfied.

From the iterative reconstruction algorithm mentioned above, each main loop of WTDM-STF algorithm is divided into three steps. These three steps are performed in the manner of alternating iteration in each of main loop. The first step is the data constraint step, which utilizes SART formula to reduce the data discrepancy between original projection data and the forward projection of the reconstructed image. The second step is the soft-threshold filtering step, which is adopted to reduce the WTD of the image. In the third step, an acceleration technique is employed to accelerate the converging speed of the WTDM-STF algorithm. From the Soft-threshold filtering step mentioned above, it is found that the pseudo-inverse of 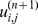 not only related to the results of soft-threshold filtration function 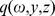, but also related to the parameter 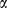. The parameter 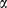 here determines the weight of the contribution of four diagonal pixels to the center pixel. By this process, the directional continuity of gradients can be effectively enforced. Thus, the edge structure information of the object can be effectively preserved and the streak artifacts can be significantly reduced.

### Data acquisitions

**Figure 1:**
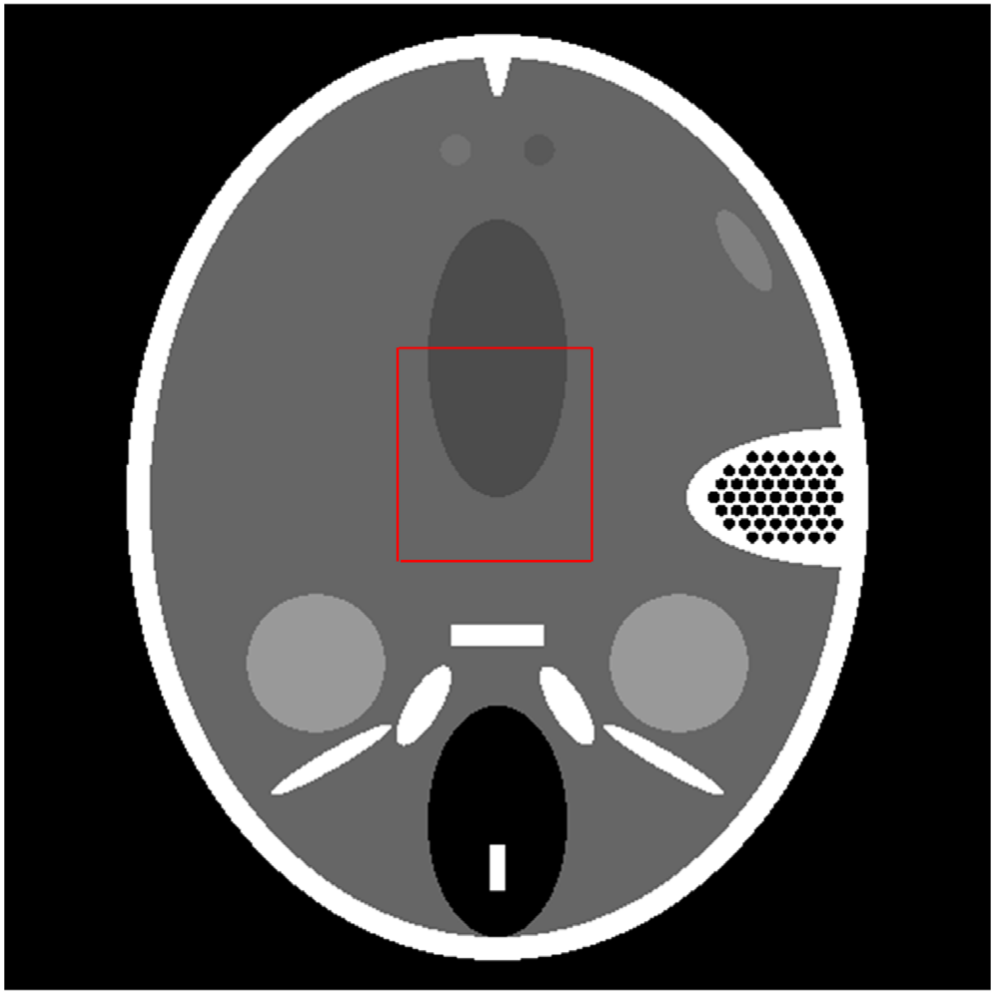
A typical slice of the FORBILD head phantom.

To demonstrate the validity of our WTDM-STF algorithm, we implemented it on a PC (2.67 GHz Intel Core i5 CPU, 4.0 G memory, Windows 7 operating system) coded in Microsoft Visual C++2008. We tested the reconstruction algorithms for few-view CT using a typical slice of low-contrast FORBILD head phantom with matrix size 512×512 [35] shown in Figure 1. Since the high-frequency and high-contrast fine inner ear structures caused severe artifacts overlapping with the low-contrast structures, more projections were used to reconstruct low-contrast structures well. In our experiments, the projection data is generated at 40 view angles specified by:
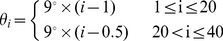
(4)


Though sparse, the angles cover 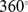 about the object. We chose a circular scanning locus of radius 51.1 cm and fan-beam geometry. The object was fixed, the X-ray source and the detector rotated around the rotation axis synchronously. The distance between source and rotation axis was set to 293.1 cm. The pixel size of the reconstructed image was 0.1×0.1 cm^2^. The equi-distance virtual detector array we used had 1025 elements, each of which had an aperture 0.05 cm. The noise-free projection data are generated by applying the system matrix to the typical slice of the FORBILD head phantom. For the noisy case, the noisy projection data are generated by adding Gaussian noise to the noise-free projection data generated previously. The standard deviation of the Gaussian noise is 0.05% of the maximum value of the projection data and the average value is zero.

### Performance evaluations

To evaluate the performance of the WTDM-STF for few-view CT image reconstruction, the following four metrics were utilized: (1) root-mean-square error (RMSE); (2) peak signal-to-noise ratio (PSNR); (3) normalized root mean square distance (NRMSD) [36]; (4) normalized mean absolute distance (NMAD) [36]:
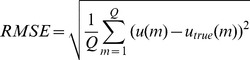
(5)

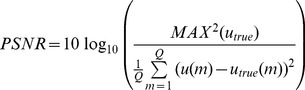
(6)

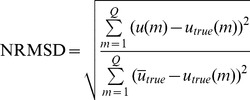
(7)

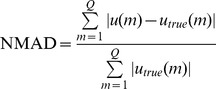
(8)where 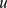 denotes the image reconstructed, 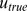 denotes the original phantom image, 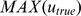 denotes the maximum density value of the original phantom image, 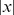 denotes the absolute value of 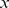, 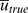 denotes the average of the densities in the interest of region (ROI) wherein 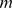 indexes the pixels in the ROI. 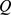 is the number of pixels in the ROI. Both the values of NRMSD and NMAD are close to 0 if the reconstruction is approximately equal to original phantom image. The NRMSD and NMAD measures emphasize different aspects of image quality. A large difference in a few places causes the value of NRMSD to be large. Note that the value of NRMSD is 1.0 if the reconstruction is a uniformly dense image with the correct average density. As opposed to NRMSD, NMAD emphasizes the importance of a lot of small errors rather than of a few large errors. Note that the value of NMAD is 1.0 if the reconstruction is a uniformly dense image with zero density.

### Statistical Analysis

Statistical analysis is performed using a user-friendly statistical software (MedCalc [37], Ostend, Belgium). To assess the performance evaluations of image quality (RMSE, PSNR, NRMSD, NMAD) presented in (5)–(8), the tests of statistical significance are performed using 500 slices of the FORBILD head phantom. First, we perform the F-test. If the associated (two-sided) P-value is less than the conventional 0.05, the null hypothesis is rejected and the conclusion is that the two variances do indeed differ significantly. If the P-value is low (P<0.05), the variances of the two samples cannot be assumed to be equal and it should be considered to use the t-test with a correction for unequal variances (Welch’s t test, [38]). The variables are expressed as Mean ± SD (standard deviations). For Welch’s t test, when the P-value is less than the conventional 0.05, the null hypothesis is rejected and the conclusion is that the two means do indeed differ significantly.

## Results and Discussion

In order to verify the superiority of our WTDM-STF algorithm, we made the comparison with the following two algorithms: (1) the classical SART algorithm and (2) the acceleration version of TDM-STF algorithm [28], respectively. For all the above reconstruction algorithms, the stopping criterion was defined as reaching the maximum iteration number 400. In all the experiments, we set the relax parameter 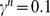 in the SART iteration formula. For all iterative reconstruction algorithms, the initial image was 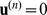.

**Figure 2:**
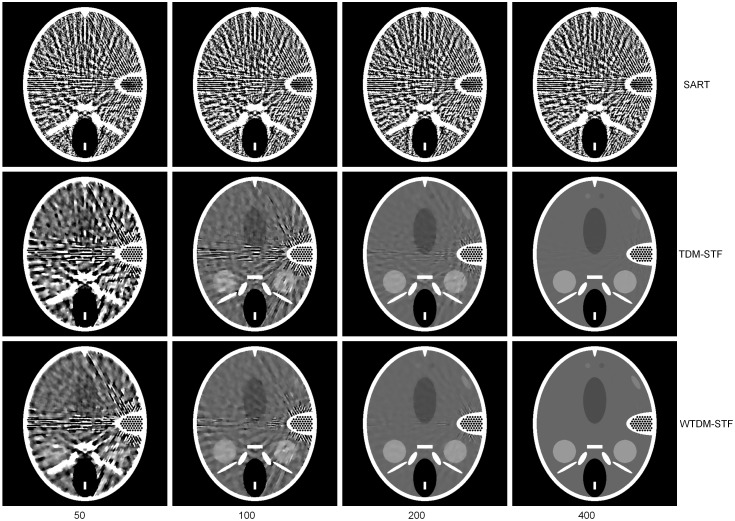
Images reconstructed by SART (first row), TDM-STF (second row) and WTDM-STF (third row) algorithms after 50, 100, 200, 400 iterations using noise-free projections, respectively. The gray scale window is set to [1.03, 1.08].

Figure 2 shows the images reconstructed by different algorithms using 40 noise-free projections. The images from up to bottom are reconstructed by SART, TDM-STF and WTDM-STF, respectively. The images from left to right are reconstructed with 50, 100, 200, 400 iterations respectively. The gray scale window is set to [1.03, 1.08]. It can be found that the images reconstructed by SART have severe streak artifacts due to the few-view projection data. While the artifacts can be better suppressed by TDM-STF and WTDM-STF after certain iterations. In the earlier iterations (such as 50 and 100 iterations), the reconstructed images are distorted nearby the high-contrast fine inner ear structures. With the increase of the iteration numbers, the streak artifacts are effectively reduced by WTDM-STF and TDM-STF. As can be seen from the first column of Figure 2, the WTDM-STF has more advantage of reducing the streak artifacts than TDM-STF. From the following two columns of Figure 2, we also come to the conclusion that the streak artifacts can be suppressed more effectively by WTDM-STF than TDM-STF. From the last column of Figure 2, it shows that when the iteration number is big enough, the reconstruction results have similar quality of vision. However, since the WTD measure enforces the gradient sparsity and the gradient continuity, the streak artifacts can be better suppressed by WTDM-STF than TDM-STF.

**Figure 3:**
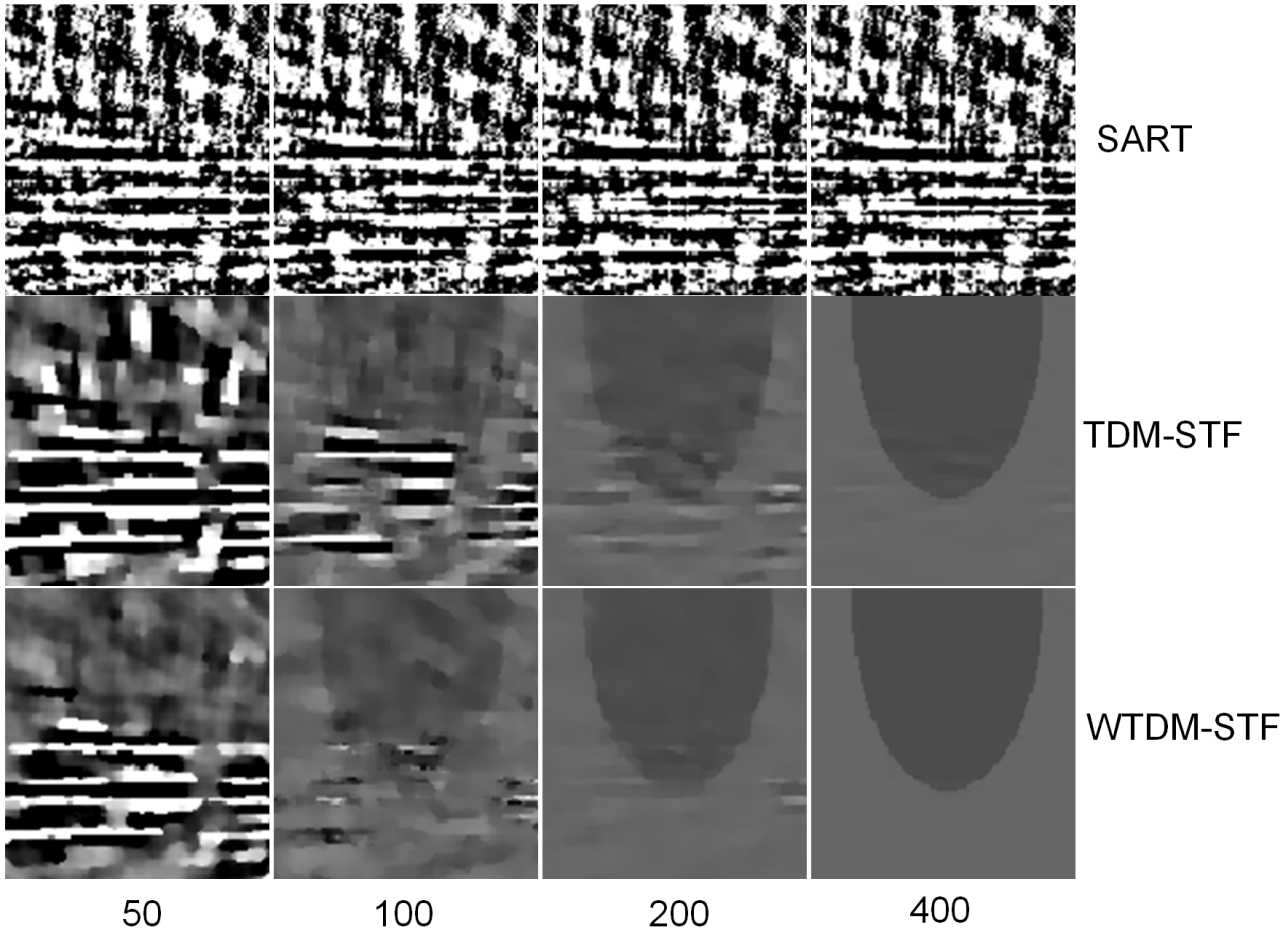
Zoom-in views of images reconstructed by SART (first row), TDM-STF (second row) and WTDM-STF (third row) algorithms after 50, 100, 200, 400 iterations, respectively. The gray scale window is set to [1.03, 1.08].

To further demonstrate the superiority of our algorithm, the zoom-in views of the images reconstructed corresponding to the selected region in Figure 1 are shown in Figure 3. It illustrates that the images reconstructed by WTDM-STF have superior image quality for different iterations. And the streak artifacts are significantly reduced by WTDM-STF and TDM-STF with the increase of iterations. Moreover, it shows that the edges are more accurate and better preserved by WTDM-STF in term of maintaining the structure information of ROI.

**Figure 4:**
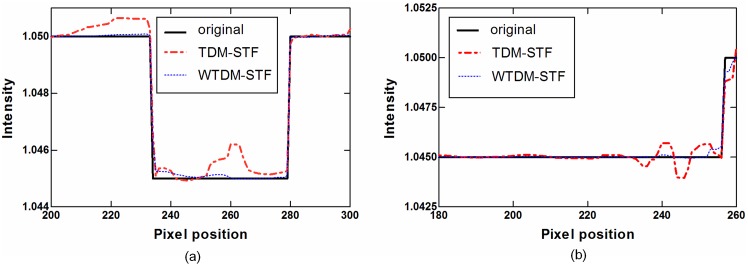
1D profiles of the images reconstructed by different algorithms using noise-free projections. (a) Horizontal profiles (240th row, 200th column to the 300th column); (b) Vertical profiles (258th column, 180th row to the 260th row).

To visualize the difference between WTDM-STF and TDM-STF, Figure 4 displays the horizontal and vertical profiles of the images reconstructed by WTDM-STF and TDM-STF. Figure 4(a) shows the horizontal profiles across the 240th row from the 200th column to the 300th column. Figure 4(b) shows the vertical profiles across the 258th column from the 180th row to the 260th row. It can be seen that the profiles of WTDM-STF match well with that from the typical slice of original phantom. The results indicate that the gains than from the WTDM-STF are more noticeable compared with those from the TDM-STF.

**Figure 5:**
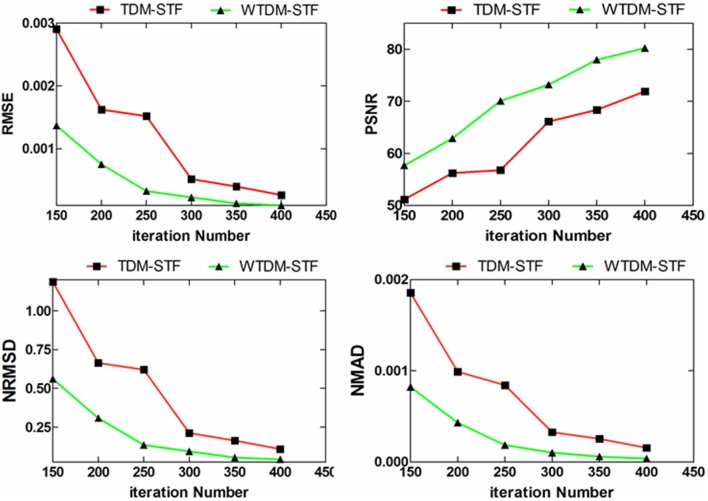
Different performance evaluations as a function of iteration numbers on the reconstructions by TDM-STF and WTDM-STF.

In addition to visual inspection of the results, Table 1 lists the RMSE, PSNR, NRMSD and NMAD measures of the images (as shown in Figure 3) reconstructed by SART, TDM-STF and WTDM-STF with 400 iterations. The quantitative results from both the TDM-STF and WTDM-STF exhibited better results than that from the SART algorithm in terms of the four measures. As can be observed from the Table 1, the WTDM-STF outperforms the TDM-STF with more than 60% gains in terms of the RMSE, NRMSD and NMAD measures and with more than 10% gains in terms of the PSNR measure. In Table 1, the WTDM-STF shows better performance since the artifacts near the edge of the object affect the accuracy of the image reconstructed. Considering the gradient sparsity and gradient continuity simultaneously in WTDM-STF, the reconstruction is more closer to typical slice of original phantom image, where the edge structure information of the object has been better preserved. In the experiments, it finds that the more the iterations, the better the image quality. Thus, to clearly demonstrate the superiority of WTDM-STF, the reconstructions after 150 iterations are considered. Figure 5 shows different performance evaluations as a function of iteration numbers on the reconstructions. It can be observed that the WTDM-STF consistently outperforms TDM-STF in these profiles.

**Table 1:** Evaluations of the results reconstructed by different algorithms (with 400 iterations from noise-free projections for a typical slice of the FORBILD head phantom).

	RMSE	PSNR	NRMSD	NMAD
SART	0.0843	21.9048	34.4883	0.0618
TDM-STF	0.000266	71.9376	0.1087	0.000155
WTDM-STF	0.000102	80.2738	0.0416	0.000037

**Figure 6:**
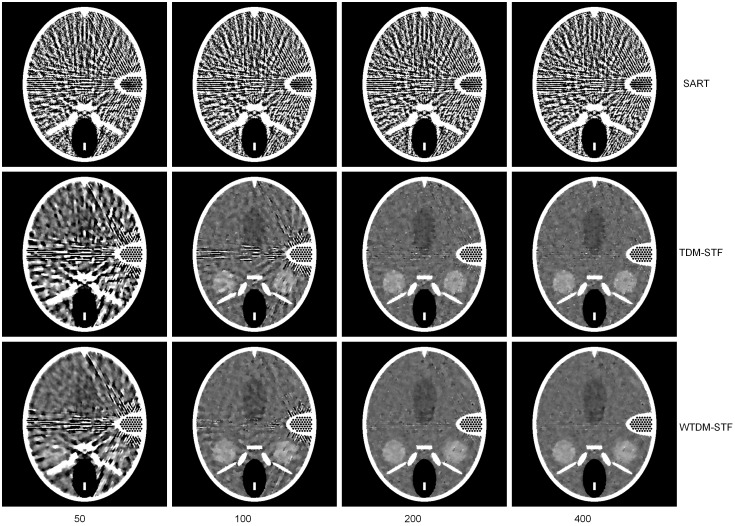
Images reconstructed by SART (first row), TDM-STF (second row) and WTDM-STF (third row) algorithms after 50, 100, 200, 400 iterations using noisy projections, respectively. The gray scale window is set to [1.03, 1.08].

**Figure 7:**
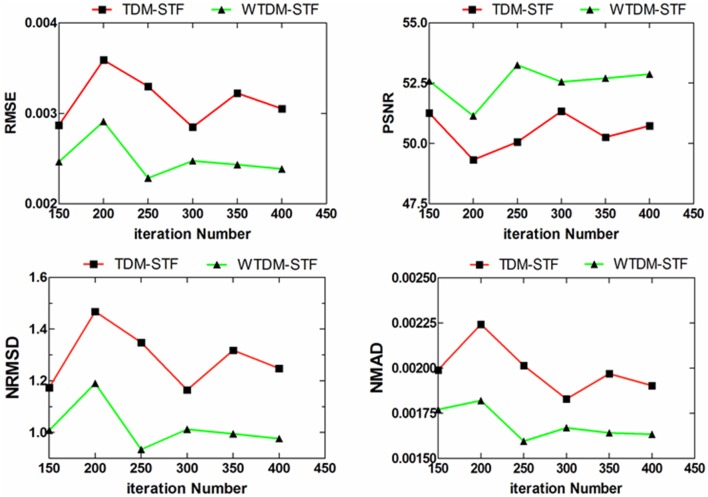
Different performance evaluations as a function of iteration numbers on the reconstructions by TDM-STF and WTDM-STF from noisy projection data.

In the practical applications, the projection data usually contains measurement noise. Then, we repeated the aforementioned experiments. Figure 6 gives the reconstructed images from noisy projection data. It can be found that the SART suffers severe streak artifacts. Compared with TDM-STF and SART, WTDM-STF causes fewer artifacts and the edges are better kept. Table 2 lists the same quantitative evaluations as in Table 1 but from noisy cases with 400 iterations. From Table 2, it shows that the results by our algorithm have better performance which are consistent with the results presented in Table 1. As can be observed from the Table 2, the WTDM-STF outperforms the TDM-STF with more than 15% gains in terms of the RMSE, NRMSD and NMAD measures and with more than 4% gains in terms of the PSNR measure. The good performance of WTDM-STF is attributed to the WTD measure which can better suppress noise and preserve the edge of the object. Figure 7 also shows different performance evaluations curves as Figure 5. It can be observed that, for noisy case, the WTDM-STF slightly outperforms TDM-STF in terms of these performance evaluations. Therefore, the WTDM-STF is a more robust algorithm for few-view CT.

**Table 2:** Evaluations of the results reconstructed by different algorithms (with 400 iterations from noisy projections for a typical slice of the FORBILD head phantom).

	RMSE	PSNR	NRMSD	NMAD
SART	0.0858	21.7519	35.1011	0.0630
TDM-STF	0.0030	50.7433	1.2467	0.0019
WTDM-STF	0.0024	52.8677	0.9762	0.0016

To further support the conclusion statements and assess the performance evaluations of image quality presented in Tables 1 and 2, we performed the tests of statistical significance using 500 slices of the FORBILD head phantom. For the experiments mentioned above, we perform the F-test first. Since the P-values are low (P<0.05), the variances of the these samples cannot be assumed to be equal. Thus, we perform the Welch’s t test. The statistical analysis results of performance evaluations of image quality between different algorithms with 100 iterations for 500 reconstruction images are summarized in Tables 3 and 4, respectively. The variables are expressed as Mean ± SD. From Table 3, there are obvious statistical differences in the values of RMSE, PSNR, NRMSD, NMAD between any two algorithms (P<0.0001). And the values of RMSE, NRMSD, NMAD by WTDM-STF are significantly lower than that of TDM-STF and SART. The value of PSNR by WTDM-STF is higher than that of TDM-STF and SART. From Table 4, the values of RMSE, PSNR, NRMSD, NMAD of WTDM-STF and TDM-STF had significant statistical difference from that of SART (P<0.0001). It shows that both WTDM-STF and TDM-STF have better performance than SART. There are significant statistical differences between WTDM-STF and TDM-STF in RMSE (P = 0.0021<0.05), NRMSD (P = 0.0006<0.05), NMAD (P<0.0001). The values of RMSE, NRMSD, NMAD show that WTDM-STF has better performance than TDM-STF. There is no obvious statistical difference in the PSNR value between WTDM-STF and TDM-STF (P = 0.0925>0.05).

**Table 3:** Summary of Welch’s t test analysis results of performance evaluations of image quality between different algorithms (with 100 iterations from noise-free projections for 500 slices of the FORBILD head phantom).

				P-value		
Item	SART (A)	TDM-STF (B)	WTDM-STF (C)	A vs. B	A vs. C	B vs. C
RMSE	0.03849±0.01882	0.002728±0.002386	0.002208±0.001087	<0.0001	<0.0001	<0.0001
PSNR	34.5921±5.3182	57.8400±4.2862	58.8675±3.0864	<0.0001	<0.0001	<0.0001
NRMSD	0.06671±0.02806	0.004786±0.003732	0.003935±0.001619	<0.0001	<0.0001	<0.0001
NMAD	0.03223±0.01181	0.001692±0.001054	0.001284±0.0006269	<0.0001	<0.0001	<0.0001

**Table 4:** Summary of Welch’s t test analysis results of performance evaluations of image quality between different algorithms (with 100 iterations from noisy projections for 500 slices of the FORBILD head phantom).

				P-value		
Item	SART(A)	TDM-STF(B)	WTDM-STF(C)	A vs. B	A vs. C	B vs. C
RMSE	0.03983±0.01872	0.004180±0.002491	0.003789±0.001351	<0.0001	<0.0001	0.0021
PSNR	34.2103±5.1016	53.7299±4.1429	54.1347±3.4225	<0.0001	<0.0001	0.0925
NRMSD	0.06913±0.02764	0.007311±0.003742	0.006676±0.001774	<0.0001	<0.0001	0.0006
NMAD	0.03487±0.01112	0.003098±0.001014	0.002659±0.0005893	<0.0001	<0.0001	<0.0001

For the experiments implemented mentioned above on a PC (2.67 GHz Intel Core i5 CPU), the average computation time per iteration of WTDM-STF, TDM-STF and SART are 2.01 s, 1.97 s and 0.95 s respectively. The average computation time of WTDM-STF, TDM-STF are longer than SART, which are due to the extra time that takes by soft-threshold filtering step. And there is small difference between the average computation time of WTDM-STF and TDM-STF. However, the parallel technique such as GPU implementation can be adopted to speed up these algorithms.

## Conclusion

To solve the problem in few-view CT image reconstruction, we present a novel iterative reconstruction algorithm based on weighted total difference minimization with soft-threshold filtering (WTDM-STF). In this algorithm, the weighted total difference (WTD) is taken as our regularization term to constrain the gradient sparsity and the gradient continuity for image reconstruction in space domain. Compared with TDM-STF algorithm in which the total difference (TD) acts as the regularization term, our WTDM-STF algorithm is more effective for few-view CT. It is inferred from the experiments that from limited projection data, our WTDM-STF algorithm shows more advantages than other algorithms in terms of image quality and convergence speed. The streak artifacts can be better suppressed and more accurate images can be generated by our WTDM-STF algorithm for few-view CT. And more edge structure information of the object can be preserved compared with the traditional ones. While for limited-angle CT, that is the projections are collected in a limited angular range less than 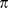, our algorithm may need to be improved since the artifacts caused by this case are more complicated than that of few-view CT. As limited-angle CT is also an important CT imaging modality for reducing the patient radiation dose, we will investigate the method for this case in the future work. For this work, while the few-view reconstruction problem was investigate only in fan-beam CT, the WTDM-STF algorithm can be extended to cone-beam CT straightforward.
